# Scaling of axial muscle architecture in juvenile *Alligator mississippiensis* reveals an enhanced performance capacity of accessory breathing mechanisms

**DOI:** 10.1111/joa.13523

**Published:** 2021-07-23

**Authors:** Kayleigh A. R. Rose, Peter G. Tickle, Ruth M. Elsey, William I. Sellers, Dane A. Crossley, Jonathan R. Codd

**Affiliations:** ^1^ Department of Biosciences College of Science Swansea University Wales UK; ^2^ School of Biomedical Sciences Faculty of Biological Sciences University of Leeds Leeds UK; ^3^ Louisiana Department of Wildlife and Fisheries Rockefeller Wildlife Refuge Grand Chenier LA USA; ^4^ Department of Earth and Environmental Sciences Faculty of Science and Engineering University of Manchester Manchester UK; ^5^ Department of Biological Sciences University of North Texas Denton TX USA; ^6^ Faculty of Biology, Medicine and Health University of Manchester Manchester UK

**Keywords:** allometry, archosaur, axial anatomy, breathing, crocodilian, flexibility, locomotion, muscle architecture, ventilatory mechanics

## Abstract

Quantitative functional anatomy of amniote thoracic and abdominal regions is crucial to understanding constraints on and adaptations for facilitating simultaneous breathing and locomotion. Crocodilians have diverse locomotor modes and variable breathing mechanics facilitated by basal and derived (accessory) muscles. However, the inherent flexibility of these systems is not well studied, and the functional specialisation of the crocodilian trunk is yet to be investigated. Increases in body size and trunk stiffness would be expected to cause a disproportionate increase in muscle force demands and therefore constrain the basal costal aspiration mechanism, necessitating changes in respiratory mechanics. Here, we describe the anatomy of the trunk muscles, their properties that determine muscle performance (mass, length and physiological cross‐sectional area [PCSA]) and investigate their scaling in juvenile *Alligator mississippiensis* spanning an order of magnitude in body mass (359 g–5.5 kg). Comparatively, the expiratory muscles (*transversus abdominis*, *rectus abdominis*, *iliocostalis*), which compress the trunk, have greater relative PCSA being specialised for greater force‐generating capacity, while the inspiratory muscles (*diaphragmaticus*, *truncocaudalis ischiotruncus*, *ischiopubis*), which create negative internal pressure, have greater relative fascicle lengths, being adapted for greater working range and contraction velocity. Fascicle lengths of the accessory *diaphragmaticus* scaled with positive allometry in the alligators examined, enhancing contractile capacity, in line with this muscle's ability to modulate both tidal volume and breathing frequency in response to energetic demand during terrestrial locomotion. The *iliocostalis*, an accessory expiratory muscle, also demonstrated positive allometry in fascicle lengths and mass. All accessory muscles of the infrapubic abdominal wall demonstrated positive allometry in PCSA, which would enhance their force‐generating capacity. Conversely, the basal tetrapod expiratory pump (*transversus abdominis*) scaled isometrically, which may indicate a decreased reliance on this muscle with ontogeny. Collectively, these findings would support existing anecdotal evidence that crocodilians shift their breathing mechanics as they increase in size. Furthermore, the functional specialisation of the *diaphragmaticus* and compliance of the body wall in the lumbar region against which it works may contribute to low‐cost breathing in crocodilians.

## Graphical abstract

The figure shows the head and upper torso of an American alligator.

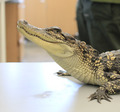

## 1 INTRODUCTION

Vertebrate trunk muscles can have multifaceted functions in locomotion, body support and respiration (Carrier, 1987; Codd et al., 2005; Farmer & Carrier, 2000a; O'Reilly et al., 2000; Schilling, 2011). Understanding the inter‐ and intra‐specific variations in the functional anatomy of the trunk can reveal adaptations that facilitate simultaneous breathing and locomotion (Brainerd & Owerkowicz, 2006; Carrier, 1991; Klein & Codd, 2010; Lambertz & Perry, 2015). Crocodilians are an interesting group in which to examine these adaptations as they have diverse breathing mechanics and locomotor modes (Codd et al., 2005; Codd et al., 2019; Farmer & Carrier, 2000a; Gans & Clark, 1976). However, the plasticity of these systems is little understood and there is currently no quantitative data on the functional anatomy of the crocodilian trunk.

Lung ventilation in crocodilians is facilitated via contractions of trunk muscles that control costal rotation, visceral displacement, pelvic rotation, vertebral flexion and translation of gastralia on the ventral surface (Claessens, 2009; Codd et al., 2019; Farmer & Carrier, 2000a; Gans & Clark, 1976; Naifeh et al., 1970). Two components of this system are ancestral. One, the sub‐costal *transversus abdominis*, is an expiratory pump, basal to tetrapods (Brainerd, 1999). It contracts bilaterally to push the liver–lung complex anteriorly and rotate the mobile and elongated pubic plates craniodorsally causing expulsion of air (Gans & Clark, 1976). The other, the costal aspiration pump, is ancestral to amniotes (Brainerd, 1999) and involves bilateral intercostal muscle contraction to rotate the tripartite ribs craniolaterally or caudomedially in inspiration or expiration, respectively (Brocklehurst et al., 2017; Gans & Clark, 1976). In the fish ancestors of amniotes, both the transversus and costal pumps functioned originally in lateral bending of the trunk for propulsion. With the evolution of the respiratory roles of these pumps came a biomechanical constraint on simultaneous breathing and locomotion for early amniotes, Carrier's constraint (Carrier, 1987, 1991). However, most extant amniote groups have evolved accessory breathing mechanisms to overcome this constraint (Brainerd & Owerkowicz, 2006; Codd & Klein, 2010; Klein & Codd, 2010). In crocodilians, breathing and locomotion are decoupled by their upright gait, derived accessory breathing muscles and transverse processes on the vertebrae that function as attachment sites for epaxial muscles, thereby reducing lateral trunk bending (Farmer & Carrier, 2000b). Crocodilians can, therefore, fine‐tune ventilatory rate with activity that is only intermittently correlated, or even uncorrelated with, the gait cycle (Farmer & Carrier, 2000a, 2000b,2000a, 2000b).

Breathing flexibility in crocodilians is facilitated by the more derived components of the respiratory system which are also the principal muscles controlling dive performance. It remains contentious, however, as to which function evolved first (Uriona & Farmer, 2006). The accessory *diaphragmaticus* is a thin muscle that encases the viscera with straps originating from the cranial aspect of the ischia and caudal‐most gastralia within the *rectus abdominis* (Boelaert, 1942; Farmer & Carrier, 2000a; Gans & Clark, 1976; Munns et al., 2012) or epipubis (Fechner & Schwarz‐Wings, 2013; Gans & Clark, 1976). Contraction of the *diaphragmaticus* pulls the liver caudad and increases thoracic volume facilitating inspiration (Farmer & Carrier, 2000a; Gans & Clark, 1976; Naifeh et al., 1970). Inspiration can also be facilitated by pubic muscles (*ischiopubis*, *ischiotruncus* and *truncocaudalis*) that rotate the pubic plates ventrocaudally to increase abdominal volume (Claessens, 2009). In expiration, the *rectus abdominis* may also be recruited to displace the gastralia cranially, push the viscera craniodorsally and rotate the pubic plates craniodorsally to expel air (Claessens, 2009; Gans & Clark, 1976). Furthermore, in forced expiration, it was recently demonstrated that the epaxial *iliocostalis* can be recruited (Codd et al., 2019). The *iliocostalis* lies with a myotomal arrangement across and between the vertebral rib elements embedding uncinate processes, which are accessory breathing structures ancestral to archosaurs (Codd et al., 2019). While costal aspiration and diaphragmatic visceral translation are the principal mechanisms for the control of tidal volume in crocodilians (Claessens, 2009; Gans & Clark, 1976; Munns et al., 2012; Uriona & Farmer, 2008), their contributions can vary greatly, and the constraints underlying this remain poorly studied. Energetic demand, body temperature, digestive state and being partially or fully submerged in water all influence muscle recruitment. However, these factors often covary in studies, making it difficult to understand their independent effects upon breathing (Codd et al., 2019; Gans & Clark, 1976; Munns et al., 2012; Uriona & Farmer, 2006).

Another variable which confounds experimental results on physiological performance is body size which can constrain functional anatomy (Schmidt‐Nielsen, 1984). The need for detailed quantitative functional anatomy of the trunk in crocodilians of different body sizes has long been noted (Gans, 1976; Munns et al., 2012). Crocodilians undergo changes in body size of several orders of magnitude in growing continuously from hatching (~30 g, 15–20 cm) and through adulthood (200–400 kg, 3m+). With increasing body size and stiffening of the trunk, muscle force demands increase disproportionately, and the basal costal aspiration pump is expected to be constrained. It was hypothesised by Munns et al (2012) that larger crocodilians, in response, may rely more upon diaphragmatic breathing. Investigating the functional specialisation of the trunk muscles and any changes during early ontogeny may shed more light on this (Fechner & Schwarz‐Wings, 2013; Gans, 1976; Munns et al., 2012). Muscle architecture (arrangement and geometric properties of the fascicles) is directly linked to performance allowing indirect inference of changes in functional output potential (Bodine et al., 1982; Gans, 1982; Gans & Bock, 1965; Lieber & Friden, 2000; Lieber & Ward, 2011; Roy et al., 1984; Sacks & Roy, 1982). For example, maximum force generation is proportional to effective physiological cross‐sectional area (Roy et al., 1984), and maximum working range and contraction velocity are proportional to fascicle length (Bodine et al., 1982; Winters et al., 2011). However, there is an inherent trade‐off between muscle strength and contractility when it comes to muscle design, which worsens with increasing body size. Muscles, therefore, tend to be specialised either to have more fibres in series (for strength) or more length to their fascicles (for contractility/power) and often demonstrate allometric scaling of their properties to meet functional demands.

Our principal objective here was to investigate the functional specialization of the trunk muscles in juvenile American alligators, *Alligator mississippiensis* and scaling of muscle properties in specimens spanning an order of magnitude in body mass (359 g–.5 kg). Similar work on the appendicular system has revealed a general trend towards a reduction in musculoskeletal capacity for terrestrial performance in the Alligatoridea, Gavialidae and Crocodylidae (Allen et al., 2010; Allen et al., 2014; Dodson, 1975; Farlow et al., 2005; Iijima & Kubo, 2019; Livingston et al., 2009; Meers, 2002). Given the decreasing functional capacity of the appendicular system as development proceeds in crocodilians and the continued need to support breathing, swimming and diving with the trunk, we hypothesised that anatomical properties of the axial system will demonstrate positive allometric growth as muscle performance keeps pace with functional demand. Any differences in scaling between different components of the system may be indicative of changes in respiratory mechanics with increasing body size. We also provide functional muscle descriptions and discuss our findings in relation to empirical evidence on muscle functions in breathing and locomotion.

## 2 METHODS

### 2.1 Specimens

Twenty‐six female juvenile *A*. *mississipiensis* cadaveric specimens (Table 1, body mass: 0.359–5.497 kg; snout‐vent length: 0.240–0.546 m; total length: 0.496–1.297 m) were acquired after the completion of unrelated studies at the University of North Texas between 2016 and 2019. Dissections were carried out on the day of euthanasia.

**TABLE 1:** Specimen body size metrics and other measurements taken

ID	Mb (kg)	SVL (m)	TL (m)	Muscle architecture	Mass of gastralia
11	0.359	0.240	0.495	Y	
109	0.592	0.280	0.593	Y	
1	0.575	0.281	0.535	Y	
110	0.810	0.288	0.625	Y	
4	0.763	0.300	0.625	Y	
105	1.125	0.320	0.695	Y	
106	0.889	0.320	0.675	Y	
104	0.889	0.323	0.704	Y	
108	0.920	0.330	0.710	Y	
103	1.042	0.336	0.723	Y	
2	1.019	0.342	0.723	Y	
102	1.125	0.350	0.753	Y	
107	0.986	0.350	0.757	Y	
3	1.200	0.355	0.745	Y	
111	1.386	0.367	0.773	Y	
101	1.533	0.370	0.790	Y	
6	1.765	0.396	0.833	Y	
112	1.840	0.407	0.848	Y	
5	2.456	0.420	0.903	Y	
10	2.817	0.450	0.950	Y	
8	2.995	0.450	0.960	Y	
9	3.611	0.470	0.995	Y	
7	3.658	0.480	1.020	Y	
L2_16	3.575	0.508	1.048	Y	
L1_9	4.552	0.514	1.092	Y	
L3_150	5.497	0.546	1.207	Y	
158	7.257	0.591	1.225		Y
58	7.484	0.597	1.270		Y
19	7.700	0.616	1.270		Y

### 2.2 Dissection and measurement protocols

The dermis was cut at the sternum and peeled around the trunk to expose the musculoskeletal anatomy. Muscles of the left side of the trunk were then examined and dissected following descriptions of Maurer (1896), Gasc (1981), Frey (1988), Murakami et al., (1991), Farmer and Carrier (2000a), and Tsuihiji (2007). Any details of the anatomy not found in the literature were recorded. Muscles were dissected sequentially: (a) *truncocaudalis*, *ischiotruncus*, *ischiopubis* (ventral aspect); (b) *obliquus externus superficialis*, *obliquus externus profundus*, *iliocostalis* (lateral aspect); (c) *rectus abdominis* (ventral aspect); (d) *diaphragmaticus*; (e) *obliquus internus* (lateral aspect); (f) *transversus abdominis* (medial aspect). In order to inspect the *transversus abdominis* and any intercostal muscles remaining on the ribs from the medial aspect, the ribs were removed from the trunk by cutting along their vertebral articulations.

Muscle mass (g, ±0.001), belly length (mm) and five fascicle lengths (mm) were recorded from each muscle using scales (Toledo XS204; Columbus, OH) and a measuring rule. Photographs were taken of the *iliocostalis* and *rectus abdominis* next to a 1 cm scale to quantify fascicle lengths using ImageJ software. Effective physiological cross‐sectional area (PCSA) was calculated for each muscle using Equation 1:

(1) $$\text{PCSA}\;\left({\text{cm}}^{2}\right)=\frac{\text{muscle mass}\;\left(g\right)}{\rho \left(1.06{\;\text{g cm}}^{‐1}\right)\times \;\text{average fascicle length}\;\left(\text{cm}\right)}$$

where *ρ* is the muscle density (Sacks & Roy, 1982). A muscle density of 1.06 g cm^‐3^ was assumed as found in mammals (Mendez & Keys, 1960) and birds (Paxton et al., 2010) and has previously been used for crocodilians (Allen et al., 2010; Allen et al., 2014) and monitor lizards (Cieri et al., 2020; Dick & Clemente, 2016).

The *rectus abdominis* has two portions (the outer, embedding gastralia, and the inner, without gastralia (Bhullar, 2009; Farmer & Carrier, 2000a; Fechner & Schwarz‐Wings, 2013)). There was no obvious separation of the muscle into two parts and so it was dissected as one unit. Gastralia were not removed from the *rectus abdominis* before muscle mass was measured. Gastralia masses measured from three specimens of a similar snout‐vent length (SVL) (Table 1) were used to estimate gastralial masses for all other specimens under the assumption that their masses scale isometrically. Whether or not gastralial mass was considered, conclusions from the results of subsequent analyses on scaling and relative muscle functional specialization were not affected and so we do not represent each graphically.

### 2.3 Scaling analyses

**FIGURE 1:**
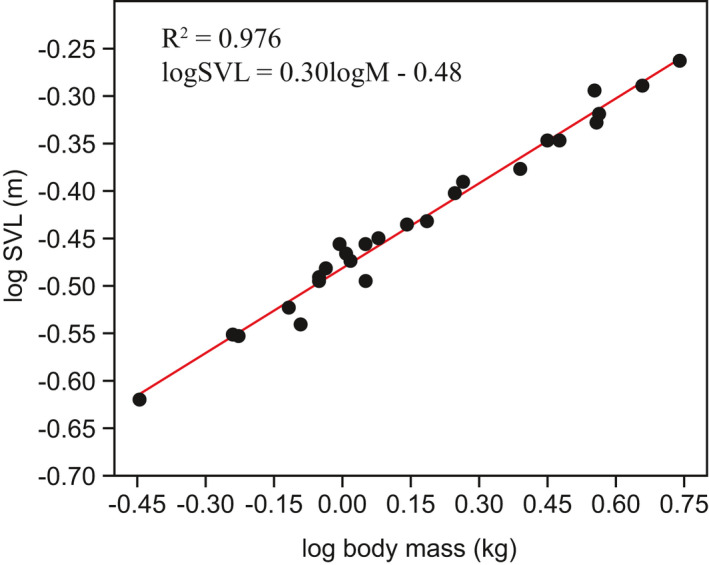
Scaling of snout‐vent length with body mass in specimens ranging 0.359–5.497 kg

Scaling of absolute muscle architectural properties with body mass was investigated using a model II regression technique (reduced major axis) in R (v 3.6.1) via the ‘lmodel2 (1.7‐3)’ package (R Core Team, 2019). In every case, log(muscle property) was regressed on log(body mass). For isometric scaling of an object, that is for an object to scale geometrically, all lengths would increase ∝ body mass^(0.33)^, all cross‐sectional areas ∝ body mass^(0.66)^ and all volumes ∝ body mass^(1.00)^. Scaling exponents and 95% CIs higher, lower or straddling these exponent values were considered representative of positive allometry, negative allometry and isometry, respectively. Body mass was chosen as the covariate rather than SVL, as SVL scaled with body mass^0.30^ (*R* ^2^ = 0.98, *p* < 0.001, 95% CIs: 0.29–0.31), with negative allometry, not isometrically (Figure 1).

## 3 RESULTS

### 3.1 Functional muscle descriptions

**FIGURE 2:**
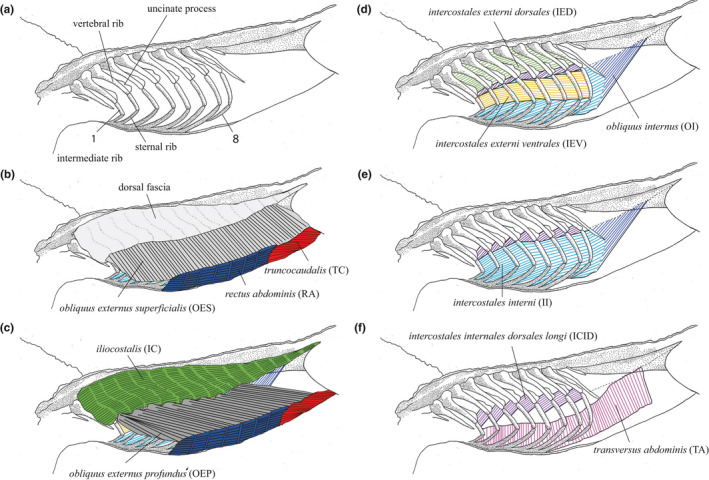
Lateral view of the anatomy of the trunk in *Alligator mississippiensis*. Anterior is to the left. a) The tripartite ribs 1–8 including the sternal (ventral), intermediate (lateral) and vertebral (dorsal) rib elements and their uncinate processes. Subsequent panels illustrate muscle layers from superficial to deep. b) The *iliocostalis* (IC) is covered by a thick fascia (the dorsal fascia) in the thoracic region which was only visible in specimens with SVL >0.40 m and body mass >1.77 kg. The *obliquus externus superficialis* (OES) attaches the ventrolateral IC, medial to the dorsal fascia; the *rectus abdominis* (RA) and the *truncocaudalis* (TC) attach to the ventrolateral rim of the OES and make up the ventral body wall. c) Following the removal of the dorsal fascia and OES, the IC lies with a myotomal arrangement across and between the verterbral ribs, while the *obliquus externus profundus* (OEP) is connected strongly to the ventral rim of the IC, dorsal lateral rims of the RA and TC. d) The *intercostales externi dorsales* and *ventrales* (IED and IEV) occupy the vertebral and intermediate intercostal spaces, respectively. The *obliquus internus* (OI) attaches to the ventral rim of the IC in the lumbar region (dotted line). e) The *intercostales interni* (II) occupy the spaces between both the sternal and the intermediate rib elements. The OI is continuous from the II. f) The *intercostales internalis dorsalis longus* (ICID) and *transversus abdominis* (TA). Notice how the TA extends dorsally only as far as the intermediate rib elements and the ICID occupy only the distal ends of the vertebral rib elements. The *transversus abdominis* (TA) is medial to the OI and also attached to the ventral rim of the IC as well as the mediolateral edge of the RA together with the eighth and floating sternal rib

**FIGURE 3:**
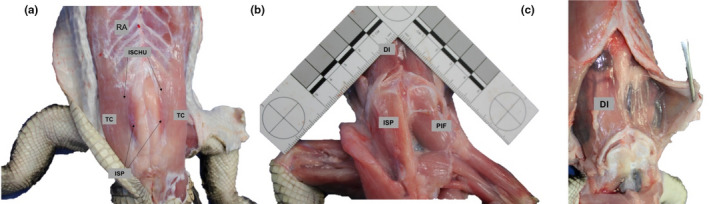
Ventral view of the muscles of the infrapubic abdominal wall in *Alligator mississippiensis*. a) The *truncocaudalis* (TC), *ischiotruncus* (ISCHU) and *ischiopubis* (ISP). The ISP was always darker in colouration and a thick fat pad always lay beneath it. b) Following removal of the TC and ISCHU. The ISP and fat pad have also been removed from the specimen's left side, revealing the *pubio*‐*ischio femoralis* (PIF). c) Origin of the *diaphragmaticus* on the last gastralial set

The tripartite ribs, including sternal, intermediate and vertebral elements with uncinate processes, and muscle names and their abbreviations are summarised in Figure 2. Uncinate processes were cartilaginous in these juvenile specimens and occurred on the vertebral rib elements 1–8. The vertebral rib elements are associated with three muscle layers: (a) superficially and in the intercostal spaces, the IC (Figure 2b,c); (b) in the intercostal spaces, the IED (Figure 2d) and (c) beneath and distally, crossing the joint between the vertebral and intermediate rib elements, the ICID (Figure 2e,f). The intermediate and sternal rib elements are surrounded by five hypaxial layers. From superficial to deep, these include: (a) OES (Figure 2b); (b) OEP (Figure 2c); (c) between adjacent intermediate rib elements only, the IEV (Figure 2d); (d) between adjacent intermediate and sternal ribs in the thoracic region, II (Figure 2e), and in the lumbar region, OI (Figure 2e) and (e) the TA and ICID (Figure 2f). On the ventral body, walls are the RA (Figure 2b,c, Figure 3a) and three muscles ventral to the pubic plates which, from superficial to deep, are the TC, ISCHU and ISP (Figure 3a,b). The DI (Figure 3c), TC, ISCHU and ISP have previously been described in detail for *A*. *mississippiensis* and are not described here (Boelaert, 1942; Farmer & Carrier, 2000a; Gans & Clark, 1976; Naifeh et al., 1971).

#### 3.1.1 Obliquus externus superficialis

The OES (Figure 2b) is immediately ventrad to the IC. The OES extends along the lateral body wall in line with the intermediate and sternal rib elements from the first thoracic rib to the end of the lumbar region. The pectoralis and TC attach superficially onto the OES at its cranial and caudal ends, respectively. A thick fat pad was stored between the pectoralis and EOS in all specimens. Deep to the pectoralis, the OES is connected to the mesosternum by aponeurosis. Fascicles of the OES run only slightly obliquely, dorsocranial to ventrocaudal. In the thoracic region, the OES attaches onto the lateral surface of the ventral IC and the medial surface of the dorsal fascia. The dorsal fascia terminates over the dorsal projections of the OES. In the lumbar region, the OES strongly attaches to the ventral aspect of the IC. The ventral margin of the OES is connected to the lateral edge of the outer portion of the RA.

#### 3.1.2 Obliquus externus profundus

The OEP (Figure 2c) is located immediately medial to the OES. The dorsal cranial aspect of the OEP extends only as far as the second set of thoracic ribs, and ventrally, it exposes the first to five thoracic sternal rib portions. Fascicles of the OEP attach to the ventral rim of the IC so that the two portions of the external obliques effectively sandwich the ventrolateral rim of the IC. The OEP attaches strongly to the lateral aspect of the inner portion of the RA and the cranial rim of the TC. Fascicles of the OEP appear longer and run more obliquely, dorsocranial to ventrocaudal, than those belonging to the superficial portion and are continuous with those of the TC.

#### 3.1.3 Iliocostalis

Syn: *iliocostalis dorsalis* (Maurer, 1896; Tsuiji, 2007); *iliocostalis dorsali* (Frey, 1988). The epaxial IC (Figure 2b,c) lies across and between the thoracic vertebral ribs with a myotomal arrangement and extends through the lumbar region to the cranial aspect of the ilium. Each myoseptum of the IC originates from the transverse processes of the vertebrae and extends caudolaterally from the ribs. Fascicles of the IC run craniocaudally between myosepta. In the smallest specimens, the myotomal arrangement of the IC was not visible and the muscle appeared thin and continuous with the hypaxial musculature. In specimens with SVL >0.40 m and body mass >1.77 kg, the IC was covered by a thick fibrous fascia (the dorsal fascia) throughout the thoracic region and the myotomes of the IC beneath were visibly conspicuous. Cartilaginous uncinate processes were embedded within the myosepta of the IC on the eight thoracic vertebral ribs and their presence and size varied between specimens. The myosepta and uncinate processes became visibly broader with increasing SVL.

#### 3.1.4 Rectus abdominis

The RA (Figure 2b,c, Figure 3a), on the ventral body wall, is interrupted by multiple sets of gastralia. Fascicles of the RA run craniocaudally between gastralial sets. The muscle is connected to the mesosternum, OES, OEP, TA, II, caudal sternal rib elements 7 and 8, OI, ISCHU, TC and pubic plates via the last gastralial set as is the DI. Both the OES and OEP are attached to the lateral aspects of the RA (outer and inner portions, respectively). The TC inserts onto the ventral surface of the RA about three sets of gastralia cranially, and the ISCHU approximately two sets cranially. The last gastralial set is attached to the pubis via a cartilaginous/ligamentous separation.

#### 3.1.5 Intercostales externi dorsales

Syn: *intercostales externus proprius* (Frey, 1988). Immediately medial to the IC lie the dorsal portions of the external intercostals, IED (Figure 2d). Fascicles of the IED also run craniocaudally between vertebral rib elements.

#### 3.1.6 Intercostales externi ventrales

The IEV (Figure 2d) lies immediately deep to the EOP and immediately ventrad of the IC. The IEV is external to the intercostal spaces between only the intermediate ribs and their fascicles run obliquely, dorsocranial to ventrocaudal, in parallel with those of the OEP and TC and perpendicular to those of the ICID.

#### 3.1.7 Intercostales interni

The II (Figure 2e) occupies the intercostal spaces between the intermediate and sternal rib portions with fascicles running cranioventral to caudodorsal.

#### 3.1.8 Obliquus internus

The OI (Figure 2d,e) is immediately deep to the external obliques occupying only the lumbar region. It is a thin muscle, continuous with the II, interrupted by only the 9th floating thoracic vertebral rib. The fascicle arrangement of the OI is very similar to that of the II and runs perpendicular to that of the external obliques. Dorsally, fascicles attach onto the ventral rim of the IC. Ventrally, the OI and the embedded sternal rib elements of thoracic ribs 7 and 8 are also connected to the inner portion of the RA at its lateral edges.

#### 3.1.9 Intercostales internales dorsales longi

Syn: *transversus dorsalis* (Murakami et al., 1991). The ICID (Figure 2f) are the deepest subcostal muscles located medially to the distal‐vertebral and proximal‐intermediate rib elements. The ICID are diamond shaped, roughly the size of one intercostal space, with fascicles running obliquely (cranioventral to caudodorsal). An aponeurotic sheath extends from either end. Ventrally, this sheath connects with the dorsal rim of the TA.

#### 3.1.10 Transversus abdominis

The TA (Figure 2f) is the deepest subcostal muscle (along with ICID). TA fascicles run along the dorsoventral axis. The TA is attached to the lateral edges of the inner RA and terminates one‐quarter of the way up the intermediate rib elements where it attaches onto an aponeurotic sheath which connects it to the ICID muscles. In the lumbar region, however, the TA also attaches to the ventral rim of the IC, immediately medial to the OI. A thin fascia separates the TA from the medial surface of the OI.

### 3.2 Muscle architecture

#### 3.2.1 Functional specialisation of inspiratory and expiratory muscles

**FIGURE 4:**
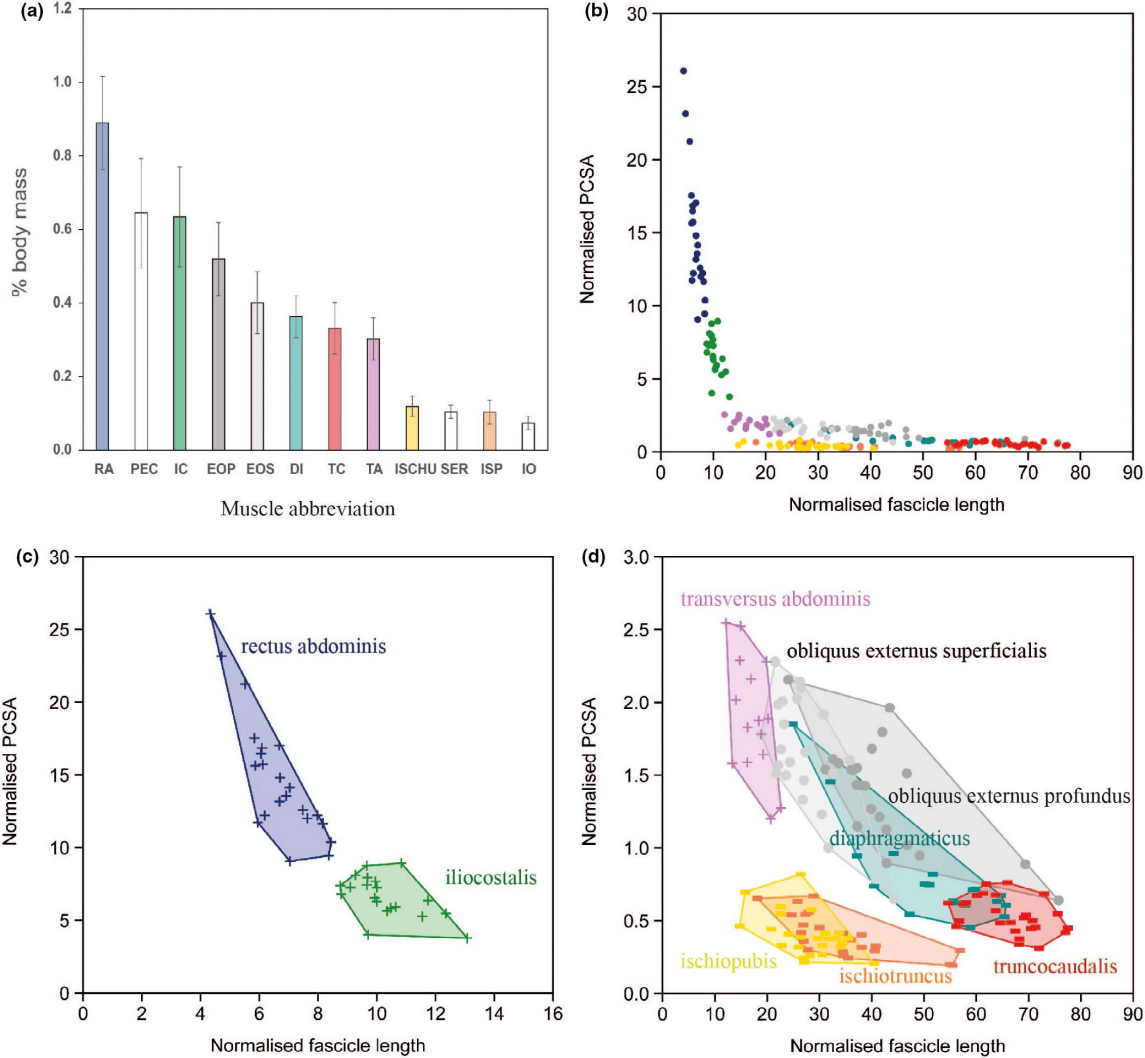
Size‐normalised trunk muscle architectural properties. a) Mean ± SD relative muscle masses. Masses of the *pectoralis* (PEC) and *serratus* (SER) are also included for comparison. b) Function space plot of axial muscles. Normalised PCSA (PCSA/body mass^(0.67)^) is plotted against normalised fascicle length (Fascicle length/body mass^(0.33)^) with one data point per muscle per individual. c) *Rectus abdominis* (RA) and *iliocostalis* (IC). d) *Transversus abdominis* (TA), *obliquus externus superficialis* (OES), *obliquus externus profundus* (OEP), *ischiopubis* (ISP), *ischiotruncus* (ISCHU), *truncocaudalis* (TC) and *diaphragmaticus* (DI). Data points for expiratory and inspiratory muscles are denoted by a ‘+’ and ‘−’, respectively. Circles represent muscles whose potential roles in breathing have not yet been investigated

Relative muscle masses are given in Figure 4a. Plots of size‐normalised PCSA versus size‐normalised fascicle length (Figure 4b–d) demonstrated that, comparatively, muscles involved in expiration (TA, RA, IC) had the greatest relative effective PCSA thereby indicating greater force‐generating capacity while inspiratory muscles (DI, TC, ISCHU, ISP) had greater relative fascicle lengths, indicative of enhanced working range and speed of contraction. The physiological functions of the EOS, EOP and IO are unknown. The external obliques are intermediate in relative mass, PCSA and fascicle lengths compared to muscles with known respiratory function indicating intermediate capacities.

#### 3.2.2 Scaling of muscle architectural properties

**FIGURE 5:**
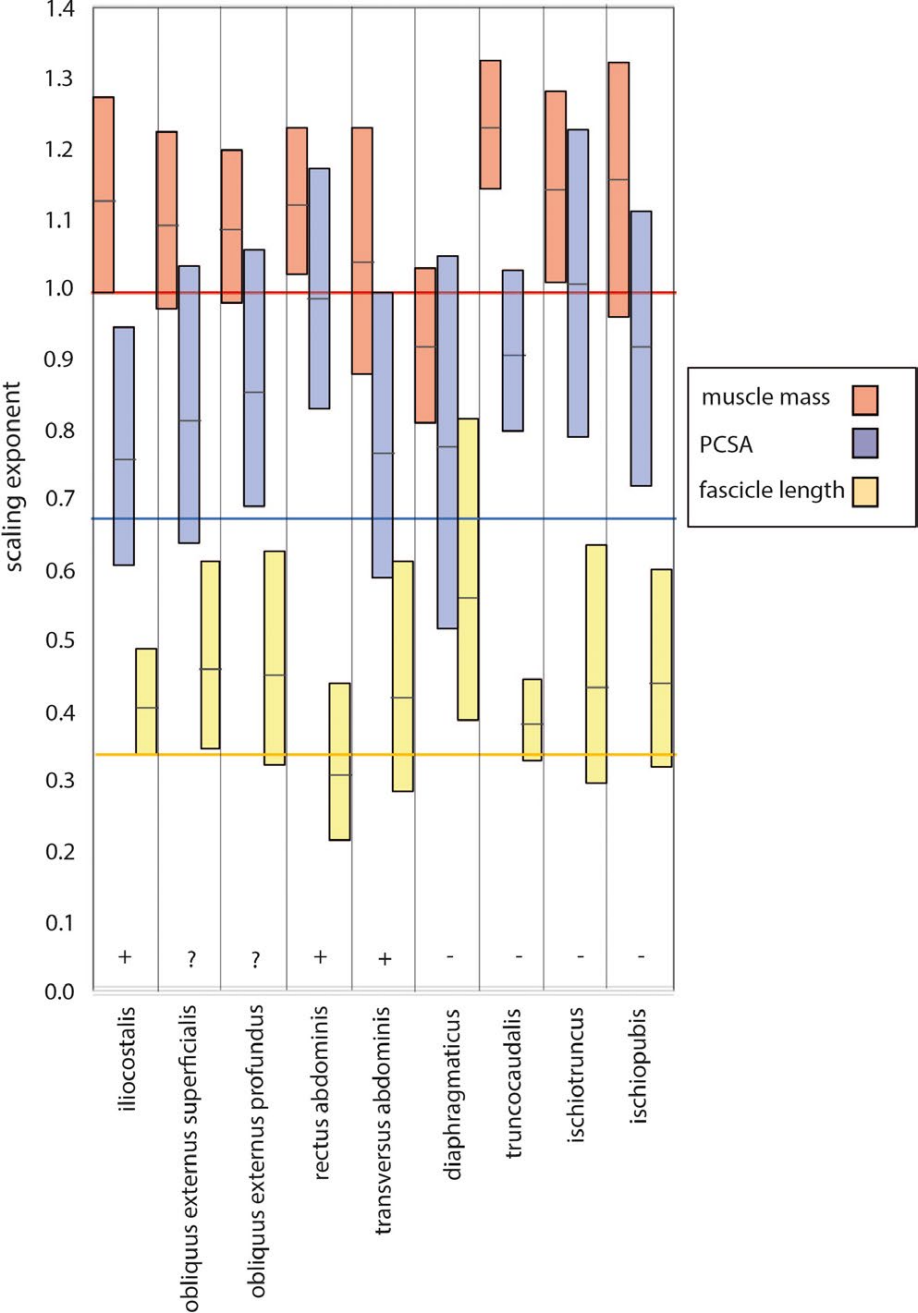
Scaling exponents for muscle architectural properties versus body mass. Boxes indicate the exponent and 95% CIs. Horizonal lines indicate exponent value for isometry in length (yellow, 0.33) PCSA (blue, 0.67) and mass (red, 1)

Scaling exponents for muscle architectural properties versus body mass is shown in Figure 5 and statistical outputs in Table 2. Muscle mass scaled with positive allometry in only the IC, RA, TC and ISCHU and scaled isometrically in the OES, OEP, TA, DI and ISP. PCSA scaled with positive allometry in the RA, TC, ISCHU and ISP, indicating a potential for increasing force generation over development. PCSA scaled isometrically in the IC, OES, TA and DI. Muscle fascicle lengths scaled with positive allometry in the IC OES, and DI, indicating potential for an increased length range over which the muscles can generate force, and capacity for speed of contraction. By comparison, fascicle lengths scaled isometrically in the EOP, TA and RA, TC, ISCHU and ISP. Therefore, positive allometric scaling was characteristic of at least one architectural property of each of the accessory breathing muscles, and only one muscle, the TA scaled isometrically in all architectural properties.

**TABLE 2:** Ontogenetic scaling exponents for logged muscle properties regressed on logged body mass (0.359–5.497 kg). Expected exponents for isometric scaling are (1) in brackets. Positive allometric growth is denoted as ‘+’ and isometric growth as ‘iso’. Subtracting estimated masses of the gastralia from the measured masses of the RA did not change the outcome of the results. All *p*‐values are two‐tailed

Muscle	Property	Slope	Lower	Upper	N	*R* ^2^	*p*	Scaling
DI	Mass	0.92 (1)	0.81	1.03	21	0.939	<0.001	iso
	PCSA	0.73 (0.67)	0.52	1.05	19	0.501	<0.001	iso
	Fascicle length	0.56 (0.33)	0.38	0.81	19	0.429	0.002	+
TA	Mass	1.04 (1)	0.88	1.23	15	0.922	<0.001	iso
	PCSA	0.77 (0.67)	0.59	0.99	15	0.805	<0.001	iso
	Fascicle length	0.41 (0.33)	0.28	0.61	15	0.560	0.001	iso
TC	Mass	1.23 (1)	1.14	1.33	26	0.969	<0.001	+
	PCSA	0.90 (0.67)	0.80	1.03	26	0.910	<0.001	+
	Fascicle length	0.38 (0.33)	0.32	0.44	26	0.864	<0.001	iso
ISCHU	Mass	1.14 (1)	1.01	1.28	25	0.922	<0.001	+
	PCSA	0.98 (0.67)	0.79	1.23	23	0.759	<0.001	+
	Fascicle length	0.43 (0.33)	0.29	0.63	23	0.226	0.022	iso
ISP	Mass	1.13 (1)	0.96	1.32	26	0.857	<0.001	+
	PCSA	0.89 (0.67)	0.72	1.11	26	0.728	<0.001	+
	Fascicle length	0.43 (0.33)	0.32	0.59	26	0.416	<0.001	iso
IC	Mass	1.13 (1)	1.00	1.27	25	0.920	<0.001	+
	PCSA	0.76 (0.67)	0.60	0.95	20	0.792	<0.001	iso
	Fascicle length	0.40 (0.33)	0.33	0.48	21	0.847	<0.001	+
EOS	Mass	1.09 (1)	0.97	1.23	25	0.97	<0.001	iso
	PCSA	0.81 (0.67)	0.64	1.03	23	0.715	<0.001	iso
	Fascicle length	0.45 (0.33)	0.34	0.61	23	0.577	<0.001	+
EOP	Mass	1.08 (1)	0.98	1.20	25	0.947	<0.001	+
	PCSA	0.85 (0.67)	0.69	1.05	23	0.778	<0.001	+
	Fascicle length	0.44 (0.33)	0.32	0.62	23	0.426	<0.001	iso
RA	Mass	1.12 (1)	1.02	1.23	23	0.959	<0.001	+
	PCSA	3.30 (0.67)	2.67	4.07	22	0.791	<0.001	+
	PCSAcorrected	3.95 (0.67)	3.38	4.62	22	8.886	<0.001	+
	Fascicle length	0.30 (0.33)	0.21	0.43	22	0.614	<0.001	iso

## 4 DISCUSSION

This study is the first investigation into the functional specialisation of the crocodilian trunk musculature. Regardless of body size, inspiratory and expiratory muscles were differentially specialised for greater relative contractile and force‐generating capacities, respectively. As hypothesised, some axial muscle properties scaled with positive allometry. In most cases, this was true for only one or two muscle properties, with muscles prioritising specialisation towards force‐generating capacity at the cost of contractility or vice versa. We place our data in context by comparison with empirical evidence (partially summarised in Table 3) on muscle activity during breathing and locomotion and how this is affected by energetic demand, prandial status and body size to illustrate biomechanical constraints.

**TABLE 3:**
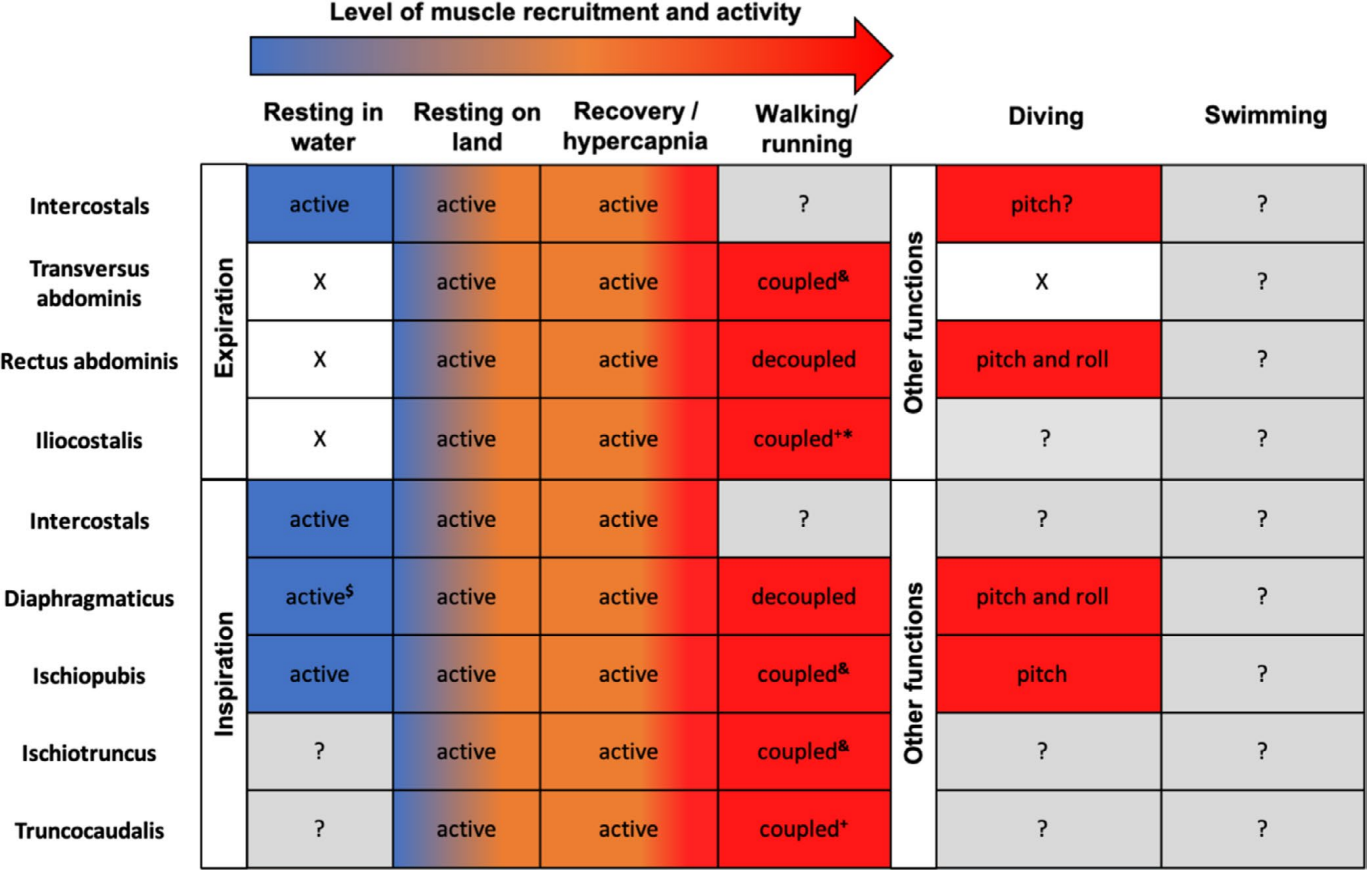
Conditions under which different muscles have been observed to be active in breathing and locomotion in crocodilians

N.B. temperature, body size and species are not considered here. Information on muscle activity was taken from Gans and Clark (1976), Farmer and Carrier (2000a, 2000b), Uriona and Farmer (2006, 2008), Uriona et al., (2009), Munns et al., (2012), Codd et al., (2019) and personal observations*.

**‘X’** muscle not active.

**‘$’** breathing can persist if the muscle is transected.

**‘&’** low intermittent activity correlated with gait cycle while principal function is in respiration, **‘+’** very high activity correlated with gait cycle.

**‘?’** has not been investigated or cannot be discerned from the literature due to differences in nomenclature and potential differences between taxa.

### 4.1 Axial musculature and the hepatic piston model of breathing

In the hepatic piston model of crocodilian breathing proposed by Gans and Clark (1976) and updated by Claessens (2009), the body walls are represented as a cylinder, which is expandable in the dorsoventral and mediolateral axes and compartmentalized into the thoracic and abdominal cavities. During inspiration, the intercostals rotate the ribs craniolaterally, the *diaphragmaticus* pulls the liver caudally and the infrapubic muscles rotate the pubic plates caudoventrally, which together draw air into the lungs and increase the volume of the cylinder (Claessens, 2009; Farmer & Carrier, 2000a; Gans & Clark, 1976). During the non‐ventilatory phase (when an inhalation is held and no axial muscles are active), the glottis is closed preventing passive exhalation via collapse of the thoracic walls (Naifeh et al., 1971) and the viscera are no longer being retracted by the *diaphragmaticus* but are kept slightly caudad and compressed by the positive pressure caused by the inspiratory air volume (Claessens, 2009). Relaxation of the glottal sphincter allows partial exhalation through passive elastic recoil of the thoracic walls and viscera (Claessens, 2009; Naifeh et al., 1971). However, experimental work suggests that the roles of the intercostals change according to surrounding media, rotating the ribs in the terrestrial environment and stabilising the ribs when submerged in water, allowing diaphragmatic breathing to take over. Furthermore, it seems that under stress, during exercise and possibly even while resting terrestrially in the absence of external hydrostatic pressure on the body, extra muscular force may be required from the abdominal walls (*transversus abdominis* and *rectus abdominis*) and dorsal epaxial musculature (*iliocostalis*) in order to speed up the caudomedial rotation of the ribs and dorsovental flattening of the cylinder to expel air (Codd et al., 2019; Gans & Clark, 1976; Uriona & Farmer, 2008).

Specialisations of the axial muscles identified in our study are therefore consistent with the hepatic piston model. There were striking differences in specialisation between expiratory and inspiratory muscles. Compared to inspiratory muscles, the expiratory muscles (*transversus abdominis*, *rectus abdominis*, *iliocostalis*) showed adaptation for increased force generation, with greater relative PCSA. The expiratory muscles are, therefore, specialised for putting the cylinder under compression to speed up the expulsion of air when necessary. Here, expiratory work is divided between the myotomes of the *iliocostalis*, gastralial rows in the *rectus abdominis* and spread through the trunk in the *transversus abdominis*, meaning greater overall forces can be exerted. It should also be noted that the force‐generating potential of the *transversus abdominis* is far exceeded by that of both the *iliocostalis* and *rectus abdominis* that have assumed accessory roles in forced expiration.

Compared to the expiratory muscles, inspiratory muscles (*diaphragmaticus*, *truncocaudalis*, *ischiotruncus*, *ischiopubis*) were specialised for a greater range of length change and speed of contraction, having longer relative fascicle lengths. A greater working range is important in the *diaphragmaticus* for the caudal displacement of the viscera in controlling up to 60% of tidal volume (Claessens, 2009). A greater working range may also be more effective for creating a negative pressure within the cylinder, allowing large volumes of air to be drawn into the thorax. The distance over which the *diaphragmaticus* can contract is also important in controlling post‐prandial vital capacity (Uriona & Farmer, 2006). The *diaphragmaticus* also plays an important role when respiratory demand increases by controlling increases the respiratory frequency and tidal volume (Farmer & Carrier, 2000a). For example, after transection of the *diaphragmaticus*, the length of time required to complete inspiration increased (Uriona & Farmer, 2006), and exercise‐induced increases in respiratory rate and tidal volume were reduced (Munns et al., 2012). Conversely, for resting animals, transection of the *diaphragmaticus* does not affect any breathing parameters (Munns et al., 2012). Together these findings support the hypothesis that costal ventilation can meet respiratory demands at rest but the *diaphramaticus* is recruited to enhance respiratory effort as metabolic demand increases oxygen demand (Munns et al., 2012).

Both external oblique muscles demonstrated intermediate masses and regional specialisations, but comparatively, the superficial portion, prioritising force generation over working range, and the deep portion, prioritising working range over force generation. We speculate that a respiratory role for these muscles seems likely given that they are directly connected to muscles of the costal, abdominal and pelvic components of the ventilatory system.

### 4.2 Muscle architecture and the metabolic cost of breathing

The metabolic cost of breathing (ml O_2_ min^−1^ kg^−1^) is reported to be low in crocodilians (~5% resting metabolic rate), on a par with birds and mammals (Skovgaard et al., 2016; Wang & Warburton, 1995). It is often considered in relation to the forces that must be generated to overcome elastic recoil of the lung, resistive forces in compressing the body wall and elastic and non‐elastic forces in expanding the body wall (Perry and Dunker 1978; Milsom & Vitalis, 1984). In many amniotes, the elastic work of breathing is inversely related to breathing frequency. In lizards, geckos and turtles, for example, high frequency, low volume breaths are the most economic breathing pattern and modulating respiratory frequency, as opposed to tidal volume is more economical in controlling ventilation rate (Milsom & Vitalis, 1984; Vitalis & Milsom, 1986). However, in studies on *A*. *mississippiensis*, where changes in breathing frequency and/or tidal volume were induced to increase ventilation rate, the opposite was found, where the metabolic cost of breathing increased with frequency, but not volume (Skovgaard et al., 2016; Wang & Warburton, 1995). Similarly, following vagotomy the duration of inspiration increases, leading to a lower cost breathing (Skovgaard & Wang, 2007).

An increased inspiratory time has been hypothesised to reduce flow resistance and therefore allow for an elevated tidal volume without higher energetic cost (Skovgaard & Wang, 2007). One alternative/additional explanation for the greater metabolic cost of modulating breathing frequency as opposed to tidal volume in crocodilians might relate to muscle architectural specialisation. The *diaphragmaticus* has the potential to contribute towards a high proportion of tidal volume (Claessens, 2009) as it is specialised for developing length change economically rather than force; for any given ventilation rate, it would be more economical for this muscle to contract over a greater distance than to contract faster and more often. Every contraction of the muscle, even if smaller, would require the muscle to be active along the full length of each fascicle involved, and the cost of muscle contraction is expected to be proportional to active muscle volume (Biewener, 2016). The *diaphragmaticus* may, therefore, assist with low‐cost breathing via both its architectural specialisation and ability to modulate contraction distance and speed for large volume and low‐frequency breathing (Farmer & Carrier, 2000b). Another potential explanation, also relating to the *diaphragmaticus*, is that this muscle does not appear vary the volume of the chest area greatly and seems to have more of an effect on the volume of the ventral lumbar region, where viscera are getting compressed and released. Therefore, the *diaphragmaticus* is not having to overcome elastic and non‐elastic forces in expanding the body wall as great as the costal aspiration pump in the thoracic region.

### 4.3 Changes in breathing mechanics with increasing body size

Recruitment of accessory breathing structures has been important in mitigating the locomotor constraints on breathing (Brocklehurst et al 2020, Codd et al 2019). With increasing body size and stiffening of the trunk, the subsequent constraints on the basal costal aspiration pump are expected to necessitate a change in breathing mechanics. Anecdotal evidence suggests a reduction in costal aspiration and increases in breathing facilitated by the *diaphragmaticus* (Gans, 1976; Munns et al., 2012). The present data provide evidence in support of the hypothesis that crocodilians become more reliant on accessory breathing mechanisms as the trunk stiffens, demonstrating that the functional capacities of the muscles are enhanced. All accessory breathing muscles demonstrated positive allometric scaling of a least one muscle architectural property, specialising force‐generating capacity over contractility or vice versa. Comparatively, all architectural properties of the *transversus abdominis* scaled isometrically indicating no increase in functional capacity to support the increased demands of a greater body mass. The function of the *transversus abdominis* in expiration is basal to tetrapods and is coupled in breathing and locomotion for some species (Brainerd, 1999). In *A*. *mississippiensis*, the *transversus abdominis* has low intermittent activity that is correlated with the terrestrial gait cycle (Farmer & Carrier, 2000a). The lack of change in this muscle's specialisation may indicate a reduction in its functional role compared to other breathing muscles over ontogeny. Active expiration is important in crocodilians, however. The roles of the *iliocostalis* and *rectus abdominis* are expected to become integral in meeting respiratory demands during terrestrial locomotion as body size increases. Large volumes of exhalation would not be able to exit the glottis fast enough by passive means. Importantly, the activity of the expiratory *rectus abdominis* is decoupled from locomotion, being uncorrelated with the terrestrial gait cycle allowing high respiratory rates (Uriona et al 2009).

In the accessory *diaphragmaticus* fascicle lengths scaled with positive allometry while mass and PCSA scaled isometrically. The shift in specialization towards greater contractile capacity at the cost of force generation in the *diaphragmaticus* would allow it to provide a greater contribution to tidal volume as the costal aspiration pump becomes constrained by heavier bony elements and lower chest wall compliance. Cinematographic evidence demonstrates that the muscle displaces viscera relatively further caudad during inspiration in larger individuals which would allow for greater relative tidal volumes (Claessens, 2009). These functional changes further support the accessory role of the *diaphragmaticus* as well as the hypothesis that larger individuals are more dependent on a respiratory role of the *diaphragmaticus* (Munns et al., 2012). Furthermore, the negative allometry of the viscera (Eme et al., 2019) indicates that the visceral mass (free of food) that the *diaphragmaticus* displaces becomes relatively lower over ontogeny. If the relative force that the *diaphragmaticus* has to apply becomes lower, the efficiency of the hepatic piston mechanism likely increases with body size.

In the accessory *iliocostalis* (Codd et al., 2019) muscle mass and fascicle lengths scaled with positive allometry indicative of enhancing contractile capacity. Although PCSA scaled isometrically in this muscle, force‐generating capacity might also be enhanced with development for two reasons. Firstly, the myosepta of the *iliocostalis* originate from the transverse processes of the vertebrae which continue to scale with positive allometry following sexual maturation (Ikejiri, 2015). Secondly, there is progressive broadening and ossification of the uncinate processes embedded within its myosepta. Therefore, the relative surface area onto which the *iliocostalis* can attach and the strength of that surface area against which it works are both expected to increase.

All accessory muscles of the infrapubic abdominal wall demonstrated positive allometric scaling in PCSA. The *rectus abdominis* has a principal role in expiration during terrestrial locomotion and elevated metabolic demand (Farmer & Carrier, 2000a). Muscle mass and PCSA scaled with positive allometry in the *rectus abdominis*, enhancing the force‐generating potential of this muscle. Similarly, *truncocaudalis* increased in both relative PCSA and mass with ontogeny, but not fascicle length, enhancing capacity to generate force to rotate the pubic plates. Increases in mass in the *rectus abdominis* and *truncocaudalis* were likely due to proliferation and/or hyperplasia of muscle fascicles as the muscle became more specialised for force generation. The *ischiotruncus* and *ischiopubis* each become relatively thicker as they become more specialised for force generation at the expense of working range. The *ischiopubis* was always observed to be a deeper red colour than the other two pubic muscles (Figure 3a), indicating that this derived part of the system probably has an important aerobic role in breathing and/or locomotion although fibre typing would be required to confirm this.

Increases in relative fascicle length and relative PCSA were also found in the superficial and deep obliques, respectively, indicating the sustained importance of their function over ontogeny; however, it is not known whether they assist breathing.

### 4.4 Changes in locomotor performance with increasing body size

Allometric scaling of trunk muscle architectural properties with increasing body size would also affect performance in aquatic locomotion. Accessory breathing muscles of crocodilians also have key roles in diving (Uriona et al., 2009). The *diaphragmaticus*, for example contracts bilaterally to shift the centre of buoyancy relative to the centre of gravity in order to control body pitch and change depth in the water column. During diving, when loads are added to the tail to counteract forward pitch, *diaphragmaticus* activity increases (Munns et al., 2012). Large increases in SVL and body mass may also require the *diaphragmaticus* to shift the viscera further caudad corresponding to our finding of an increase in relative fascicle length. If the *diaphragmaticus* is transected, dive duration decreases, indicating the muscle's role in increasing the duration of dives (Uriona et al., 2009). Submerged lung volume (which would influence total mass‐specific oxygen stores) has a scaling exponent greater than that for resting metabolism in crocodilians (Wright & Kirshner, 1987), which has been interpreted as indicating that the duration of dives could increase with body size (Cott, 1961). The positive allometry in fascicle length of the *diaphragmaticus* would also support increased dive durations with increasing body size.

The *ischiopubis* is also used in controlling dive pitch. Relative force‐generating capacity of the *ischiopubis* increases with body mass, which is not consistent with its specialization to rotate the pubic plates through large moment arms; however, it is not known how pubic plate morphometrics change over ontogeny and this may affect the muscle moment arms which could mitigate this architectural change. The increase in relative force‐generating capacity may also be necessary to work against forces exerted by other trunk muscles during locomotion as body size increases.

The *rectus abdominis* is active bilaterally in controlling pitch and unilaterally in body rolling during dives. The enhanced force‐generating potential of this muscle would facilitate pitch and roll of the heavier and longer body. It may become particularly important for “death rolling” while holding heavier prey with the snout during feeding (Fish et al., 2007).

There is a paucity of evidence for exactly which trunk muscles are recruited during crocodilian swimming, which is principally powered by the tail. However, the overall mechanical efficiency of swimming in crocodilians decreases with ontogeny (Seebacher et al., 2003). If the *transversus abdominis* plays an important role in trunk bending and stabilization as it does in the salamander (Bennett et al., 2001) isometric scaling of both fascicle lengths and PCSA may pose a constraint on function. Furthermore, positive allometric scaling was more prevalent for muscle PCSA than for muscle fascicle length across the trunk muscles examined. Prioritising muscle force over contractility may be a biomechanical explanation for why crocodilians are relatively slow swimmers compared to semi‐aquatic mammals as was hypothesised by Seebacher at al (2003).

## 5 CONCLUSIONS

Here we demonstrate that the functional specialisation of the crocodilian trunk changes to meet changing biomechanical demands and constraints. We propose that ontogenetic changes in muscle architectural specialisation facilitate fine control over inspiratory volume and frequency to minimise the cost of breathing and allow sustained simultaneous breathing and locomotion. We suggest that isometric scaling of muscle properties of the *transversus abdominis* indicates that, with increasing body size, the basal tetrapod expiratory pump becomes less important compared to the derived components of the ventilatory apparatus which scale with positive allometry in some of their geometrical properties for improved contractile or force‐generating capacities. Future research directly linking anatomical changes in muscle architecture to EMG studies of muscle activity patterns across the full‐size range of crocodilians would allow us to test these ideas.

## AUTHOR CONTRIBUTIONS

JRC and PGT conceived the initial idea and performed the pilot data studies. KARR, WIS, PGT, JRC and DAC designed the study. RME facilitated alligator egg collection. KARR was responsible for data collection, analysis and interpretation and drafted the manuscript. All authors contributed to the critical revision of the manuscript.

## Data Availability

all data are included in the manuscript
